# Identification of novel mutations of Insulin Receptor Substrate 1 (IRS1) in tumor samples of non-small cell lung cancer (NSCLC): Implications for aberrant insulin signaling in development of cancer

**DOI:** 10.1590/1678-4685-GMB-2017-0307

**Published:** 2019-02-25

**Authors:** Gokhan Gorgisen, Fatma Zehra Hapil, Ozlem Yilmaz, Zafer Cetin, Suray Pehlivanoglu, Irem Hicran Ozbudak, Abdullah Erdogan, Osman Nidai Ozes

**Affiliations:** 1 Van Yuzuncu Yil University Van Yuzuncu Yil University Faculty of Medicine Turkey Van Yuzuncu Yil University, Faculty of Medicine, Medical Biology, Van, Turkey; 2 Izmir Biotechnology and Genome Center Izmir Biotechnology and Genome Center Izmir Turkey Izmir Biotechnology and Genome Center, Izmir, Turkey; 3 Akdeniz University Akdeniz University Faculty of Medicine Antalya Turkey Akdeniz University, Faculty of Medicine, Medical Biology and Genetic, Antalya, Turkey; 4 Sanko University Sanko University Faculty of Medicine Gaziantep Turkey SANKO University, Faculty of Medicine, Medical Biology, Gaziantep, Turkey; 5 Necmettin Erbakan University Necmettin Erbakan University Faculty of Science Konya Turkey Necmettin Erbakan University, Faculty of Science, Konya, Turkey; 6 Akdeniz University Akdeniz University Faculty of Medicine Antalya Turkey Akdeniz University, Faculty of Medicine, Pathology, Antalya, Turkey; 7 Akdeniz University Akdeniz University Faculty of Medicine Antalya Turkey Akdeniz University, Faculty of Medicine, Chest Surgery, Antalya, Turkey; 8 ALTAY Biopharma ALTAY Biopharma San BrunoCA USA ALTAY Biopharma, San Bruno, CA, USA

**Keywords:** IRS1, NSCLC, insulin signaling, lung cancer, IRS proteins

## Abstract

Lung cancer is the leading cause of cancer-related death, and NSCLC constitutes nearly 85%–90% of all cases. The IRS proteins function as adaptors and transmit signals from multiple receptors. Upon binding of insulin to the insulin receptor (IR), IRS1 is phosphorylated at several YXXM motifs creating docking sites for the binding of PI3Kp85, which activates AKT kinase. Therefore, we thought that gain of function mutantions of IRS1 could be related to development of lung cancer. In line with this, we wanted determine whether the IRS1 gene was mutated in the coding regions surrounding YXXM motifs. We sequenced the coding regions surrounding YXXM motifs of IRS1 using tumor samples of 42 NSCLC patients and 40 matching controls and found heterozygote p.S668T mutation in nine of 42 samples and four of nine also had the p.D674H mutation. We generated IRS1 expression vectors harboring p.S668T, p.D674H and double mutants. Expression of the mutants differentially affected insulin-induced phosphorylation of IRS1, AKT, ERK, and STAT3. Also, our mutants induced proliferation, glucose uptake, inhibited the migration of 293T cells and affected the responsiveness of the cells to cisplatin and radiation. Our results suggest that these novel mutations play a role in the phenotype of lung cancer.

## Introduction

Lung cancer is divided into two major groups: non small cell lung cancer (NSCLC) and small cell lung cancer (SCLC). About 85% to 90% of lung cancers diagnosed are NSCLC, and NSCLC comprises three main subtypes including squamous cell carcinoma, adenocarcinoma and large cell carcinoma (Cappuzzo *et al.*, 2004; Lynch *et al.*, 2004; Taron *et al.*, 2005).

The IRS proteins (IRS1-4) are the family of adaptors regulating metabolic and mitogenic signaling pathways (Hanke and Mann*,* 2009; [Bibr B13] Metz *et al.*, 2011). Following insulin binding, the insulin receptor (IR) autophosphorylates itself and creates docking sites for IRS proteins. Upon binding to IR, IRS1 is phosphorylated by IR at YXXM motifs, and binding of PI3K-p85α to pYXXM motifs activates PI3K, which results in activation of AKT (Sesti *et al.*, 2001; Ma *et al.*, 2006).

In addition to binding to the insulin receptor, IRS1 also binds to and transmits signals from the receptors of prolactin, growth hormone (GH), leptin, vascular endothelial growth factor (VEGF), tropomyosin receptor kinase B (TrkB), anaplastic lymphoma kinase (ALK), insulin like growth factor (IGF1), and integrins (Vuori and Ruoslathi*,* 1994; Senthil *et al.*, 2002; Gibson *et al.*, 2007; Chan and Lee*,* 2008; Porter *et al.*, 2012). Since these receptors induce cell proliferation, survival and migration, it was suggested that IRS1 may be involved in the development of cancer and metastasis. In line with this, the expression and phosphorylation of IRS1 has been determined in numerous cell lines, and trends toward increased expression or phosphorylation of IRS1 have been reported in many cancers (Bergman *et al.*, 1996; Rocha *et al.*, 1997; Schnarr *et al.*, 2000; Hoang *et al.*, 2004; Koda *et al.*, 2005; Han *et al.*, 2006; Ravikumar *et al.*, 2007; Sisci *et al.*, 2007)

The function of IRS1 is regulated by its expression and post-translational modifications. Its tyrosine and serine/threonine phosphorylations promote or inhibit insulin signaling, respectively (Zick 2001, 2005; Luo *et al.*, 2007; Boura-Halfon and Zick*,* 2009), although, Reiss *et al.* (2001) showed that serine phosphorylation of IRS1 increased adhesion, and decreased the motility of LNCaP cells. In lung cancer cells, silencing of IRS1 caused proliferation and induced phosphorylation of AKT (Antoniades *et al.*, 1992; Han *et al.*, 2006; Houghton *et al.*, 2010). These results suggest that IRS1 can function as growth regulator in cancer depending on the cell of origin. Additionally, genomic changes of IRS1 have been linked to development of cancer (Carvalheira *et al.*, 2003; Heather *et al.*, 2014). Therefore, we sought to determine whether IRS1 is mutated in NSCLC, and found two novel mutations in nine of 42 samples. Of these, an S668T mutation was heterozygous in nine samples, and a D674H mutation was heterozygous in four of these nine samples. The presence of these mutations in the vicinity of PI3Kp85 binding sites encouraged us to think that these mutations may affect insulin-mediated cell proliferation, migration and glucose uptake. Therefore, we generated IRS1 expression vectors harboring these mutations, transiently expressed them in 293T and A549 cells, and examined the effects of these mutations on insulin-induced proliferation, migration, glucose uptake, phosphorylations of IRS1, ERK and AKT in 293T cells. We also determined the impact of these mutations on the cisplatin-induced death of 293T, H1299, PC14, HeLa and PC3 cells**.**

## Materials and Methods

### Cell culture and reagents

Monoclonal anti-IRS1, ERK1/2, pERK1/2, AKT, pAKT and anti-phosphotyrosine were from Santa Cruz Biotechnology (Santa Cruz, CA); anti-β-Actin was from Sigma (Saint Louis MO); anti-rabbit/mouse HRP was from BioRad (Hercules, CA). All cells were grown in DMEM supplemented with 10% FBS, 100 μg/mL penicillin, 50 μg/ml streptomycin, and 1 mM glutamine.

### Western blotting

Western blots were performed according to Ozes *et al.* (1999, 2001). Briefly, 293T cells were transiently transfected with expression vectors of IRS1 for 24 h, serum starved for 16 h and treated with insulin for 5 and 30 min. Cellular lysates were prepared and 100 μg of proteins were fractionated by 10% SDS-PAGE. Blots were first labeled with anti-phosphospecific antibodies, then stripped and re-probed with the relevant non-phospho specific antibody. To determine the fold induction of phosphorylation, we determined densitometric values of phospho and total protein bands, and divided the values of phospho forms to that of total protein. To determine the relative abundance of IRS1, ERK, AKT and STAT3 we divided the densitometric values of these to that of beta-actin. Western blots were performed in triplicate.

### Tissue procurement

Fourty two tumor and 40 matching control tissues from the same patients were provided by Department of Chest Surgery of Akdeniz University, Faculty of Medicine. The experiments were undertaken with the understanding and written consent of each subject,the study methodologies conformed to the standards set by the Declaration of Helsinki, and the study methodologies were approved by the Akdeniz University Ethics Committee.

### Mutational analyses of PI3K binding sites of IRS1 in lung tissues

Genomic DNA was isolated using a Macherey-Nagel extraction kit. Genetic analysis of DNA covering PI3K-binding sites of IRS1 was performed by PCR. The following primers were used: Primer 1/1 (forward, 5’ggaggtgg cagtggaggccgactgcc3’; reverse, 5’cctcagggccgtagtagcag tc3’) Primer 1/2(forward, 5’ctggagcccagccttccacatc3’; reverse, 5’ccctgggcaggctcacctcctc3’). PCR was performed in a total volume of 25 μL, containing 1x Qiagen *Taq* polymerase buffer (Qiagen, Germany), 2 mM MgCl_2_, 6 mM dNTPs, 0.5 μM of each primer, 0.2 units Qiagen *Taq* DNA polymerase and 50 ng genomic DNA. PCR conditions were 5 min at 94 °C, followed by 35 cycles of 94 °C for 30 s, 58 °C for 1 min, 72 °C for 45 s, and one step of 72 °C for 10 min. PCR products were purified using a PCR Purification Kit (Invitrogen Carlsbad, CA), and the Big dye-terminator sequencing kit (Applied Biosystems, Foster City, CA) was used during amplification. Sequencing fragments were analysed by using an ABI Prism 3130 DNA analyzer (Applied Biosystems). Sequence chromatograms were analyzed by Finch TV.

### Transfections

Approximately 70% confluent cells were transfected with mock or IRS1 expression vectors by the calcium-phosphate precipitation method. Ectopic expression of mutant IRS1 proteins was determined by western blotting.

### Site-directed mutagenesis

Ser668 and Asp674 of human IRS-1 was mutated to Thr (S668T) and His (D674H) with the *Pfu* polymerase (Thermo Sci, USA) using primers F1.5’-acatgatgatgtcccc caccggtggctgc-3’, F2.5-’gcagccaccggtgggggacatcatcat gt-3’ R1.5-’cggtggctgctctcctcacattggaggtg-3’. R2.5’-cacctccaatgtgaggagagcagccaccg-3’. PCR conditions were 30 s at 95 °C, followed by 18 cycles of 95 °C for 30 s, 55 °C for 1 min, 72 °C for 11 min , and one step of 72 °C for 10 min. Mutations were verified by DNA sequencing.

### Cell viability testing

Cell viability was determined using an MTT assay. The cells were plated at a density of 3,000 cells/well in 96-well plates with 6 replicates, cultured in DMEM, and the next day cells were treated 100 ng/mL insulin for 72 h. Then 20?μL of MTT solution (5?mg/mL) was added for 4 h at 37 °C, medium was removed and DMSO (100?μL) was added. The plates were shaken at 600 rpm for 5 min and the absorbance of developed color was determined at 540 nm, with 690 nm as the reference wavelength.

### Glucose uptake assay

Glucose uptake was measured using a Glucose Uptake Assay Kit (Cayman Chemicals). A549 cells were transfected with vectors for 24 h, trypsinized and replated at a density of 5,000 cells/well in 96-well plates in triplicates. The cells were incubated in DMEM overnight, washed, and incubated with glucose-free medium containing glucose analog 2-NGBD in the presence or absence of insulin (100 ng/mL) in the dark. Then, the medium was removed, cells were washed with PBS, and 2-NBDG taken up by the cells was detected using fluorescent filters with excitation at 485 nm and emission at 535 nm. Fluorescein intensity was calculated by using Image J software.

### Migration assay

Migration assays were performed in duplicate by using BD Biocoat Insert Systems, USA with 8 micron pore size. Five thousand cells were plated in the upper chamber in duplicates in serum-free DMEM, the bottom wells had serum-containing DMEM. After overnight incubation, medium in upper chamber was removed, cells were fixed with methanol, and cells in the upper chamber were scraped off with cotton swab. Migrated cells were stained with hematoxylin and eosin. Images of migrated cells were obtained with Olympus IX51.

### Radiotherapy and cisplatin treatments

In total, 25,000 293T, 10,000 PC14, PC3, HeLa and 8,000 H1299 cells were seeded in 96-well plates, transfected with 300 ng plasmid and 0.4 μL lipofectamine 2000. After 24 h, cells were treated with 3 μg/mL cisplatin, and further incubated for 72 h. For radiation treatment, 293T cells transfected using the same protocol were treated with 5 Gy radiation for 10 min, and further incubated for 48 h. Cell viability was determined using an MTT assay.

### Statistical analysis

ANOVA and Kruskal Wallis tests were used to compare the data. All statistical tests were two-tailed, and a *p*-value < 0.05 was considered statistically significant.

## Results

### IRS1 mutations in YYXM surrounding regions

Since we hypothesized that mutations at or around the YXXM motifs of IRS1 may impact insulin signaling and the phenotype of lung cancer, we isolated the genomic DNA from 42 tumor and 40 normal lung tissues of NSCLC patients and sequenced the coding region of IRS1. Sequence analyses revealed a heterozygote p.S668T mutation in nine samples and a p.D674H change in four of nine patients (Figure S1b). We also detected four previously identified SNPs (Table 1, Figure S1a). The mutations we identified were adjacent to PI3Kp85 binding sites (Y612,632,662). To explore the functional significance the mutants, we generated S668T, D674S and their combinations using a flag-tagged human IRS1 expression vector (Figure S1c).

**Table 1:** Clinical features of NSCLC patients with sequence results of IRS1 gene (SCC: Squamous cell carcinoma, ADC: Adenocarcinoma, LCC:Large cell carcinoma, ADC: Adenosquamous carcinoma ; M: Male, F:Female, WT: Wild Type)

Patients Number	Gender	Age	Tumor type	Tumor size	TNM	Stage	Sequencing result
2	M	54	SCC	8 cm	T2N0M0	1b	WT
3	M	56	ASC	8 cm	T2N1M0	2a	WT
7	M	48	ASC	11 cm	T2N1M0	2b	WT
8	M	66	SCC	10 cm	T2N0M0	1b	WT
9	M	47	SCC	2 cm	T2N1M0	2b	WT
11	M	69	SCC	8.5 cm	T2N1M0	2b	**rs138975702**
13	M	67	SCC	4 cm	T3N0M0	2b	**p.S668T**
14	M	53	SCC	8 cm	T2N0M0	1b	WT
15	M	79	SCC	2 cm	T2N0M0	1b	WT
16	M	65	SCC	9 cm	T2N1M0	2b	**p.S668T, p.D674H**
18	F	69	SCC	3.5 cm	T2N1M0	2b	WT
19	M	56	ASC	7.5 cm	T2N0M0	1b	**p.S668T, p.A642S**
22	M	77	SCC	6 cm	T2N2M0	3a	WT
24	M	50	SCC	6 cm	T2N0M0	1b	WT
25	M	67	SCC	5 cm	T2N1M0	2b	WT
26	M	67	ASC	5 cm	T2N0M0	1b	**p.S668T, H599A**
27	M	68	LCC	2 cm	T1N1M0	2a	**p.S668T**
28	M	71	ADC	9 cm	T2N1M0	2b	WT
30	M	58	ASC	6.5 cm	T2N0M0	1b	WT
31	M	60	ASC	5 cm	T2N0M0	1b	WT
32	M	62	SCC	8.5 cm	T2N0M0	1b	WT
33	M	65	ASC	9.5 cm	T3N0M0	2b	**rs138975702**
34	M	62	SCC	2 cm	T1N0M0	1a	**rs143032259**
36	M	57	SCC	7 cm	T2N0M0	1b	WT
37	M	62	SCC	8.5 cm	T2N1M0	2b	WT
38	M	51	SCC	3 cm	T2N1M0	2b	WT
40	M	45	SCC	5.5 cm	T2N0M0	1b	**p.S668T, p.D674H**
42	M	57	SCC	6 cm	T2N0M0	1b	**p.S668T, p.D674H, p.A642S**
43	M	59	ADC	3.2 cm	T3N1M0	3a	WT
44	M	68	SCC	3.8 cm	T2N0M0	1b	WT
46	M	71	SCC	6.5 cm	T1N0M0	1a	WT
47	M	67	SCC	4 cm	T2N1M0	2b	**p.S668T, p.D674H**
49	M	54	SCC	3.5 cm	T2N1M0	2b	WT
53	M	68	SCC	4.2 cm	T3N0M0	2b	WT
55	M	59	SCC	2.5 cm	T1N0M0	1a	WT
56	M	55	SCC	4.5 cm	T2N1M0	2b	WT
58	M	61	ADC	2.5 cm	T2N0M0	1b	WT
59	M	54	ADC	5 cm	T2N0M0	1b	WT
60	M	63	SCC	2.5 cm	T2N1M0	2b	**p.S668T**
61	M	60	SCC	3.8 cm	T2N0M0	1b	WT
62	M	39	ADC	4 cm	T2N0M0	1b	WT
63	M	63	SCC	3 cm	T2N1M0	2b	**rs138975702**

### Effects of S668T, D674H and double mutants on insulin-induced phosphorylation of IRS1, ERK, AKT, and STAT3

**Figure 1:**
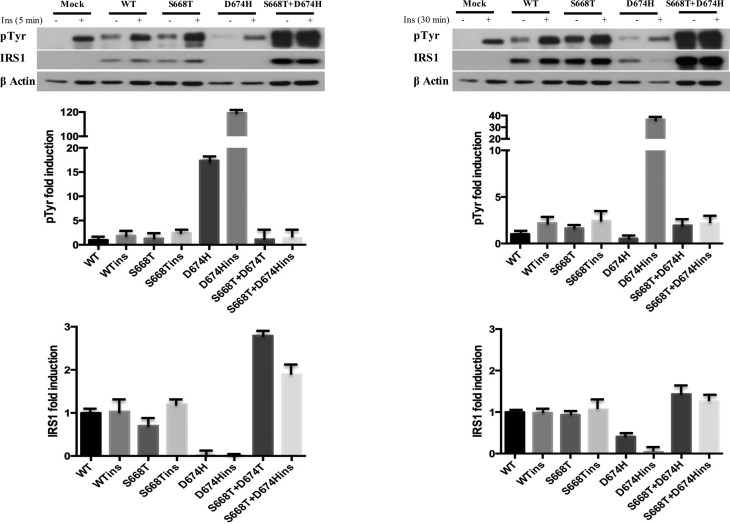
Insulin-induced tyrosine phosphorylation and expression level of IRS1 is altered by S668T and D674H mutants in 293T cells. One hundred μg of total protein was fractionated by 10% SDS-PAGE. Blots were first labeled with anti-phospho-specific antibodies, stripped and reprobed with non-phospho-specific antibody. Fold induction of phosphorylation was determined by dividing densitometric values of the phospho- band by that of total protein bands. Relative abundance of IRS1 was determined by dividing the densitometric values of IRS1 to that of beta-actin.

To determine the impact of our mutants on insulin signaling we transfected the vectors into 293T cells for 24 h, and the cells were then serum-starved and treated with insulin (100 ng/ml) for 5 and 30 min. Five minute insulin treatment induced tyrosine phosphorylation of IRS1, and this was similar in wild type and S668T mutant expressing cells. However, the level of expression of IRS1 harboring the D674H mutation was bearly detectable, although tyrosine phosphorylation was evident. When we normalized the expression of the D674H mutant to that of phosphorylation, insulin induced > 100 fold the tyrosine phosphorylation of the D674H mutant. However, in the double mutant, the stability of IRS1 increased, and the level of tyrosine phosphorylation decreased compared to the D674H mutant. The level for the D674H mutant of IRS1 was higher in cells treated with insulin for 30 min, therefore, fold induction of tyrosine phosphorylation dropped from 120 to 40 fold. These results indicate that the D674H mutation destabilizes IRS1, and this was reversed by S668T (Figure 1).

**Figure 2:**
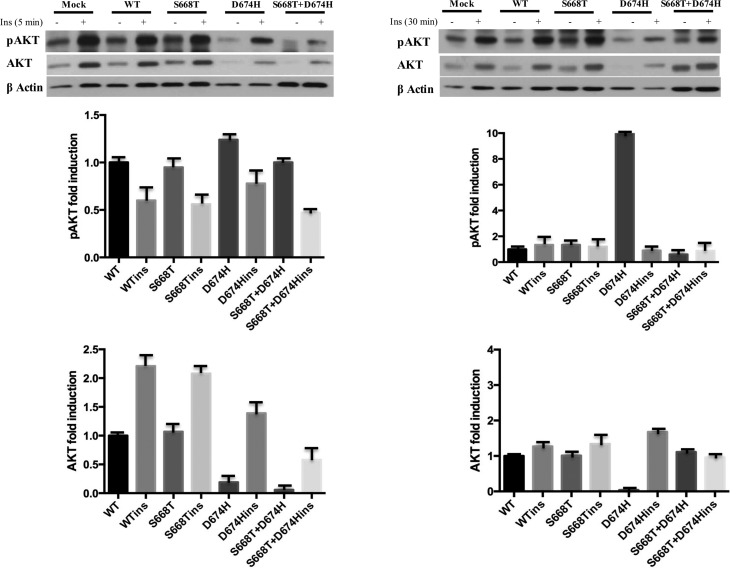
Insulin-induced phosphorylation and expression level of AKT is altered by S668T and D674H mutants in 293T cells. One hundred μg of total protein was fractionated by 10% SDS-PAGE. Blots were first labeled with anti-phospho-specific antibodies, stripped and reprobed with non-phospho-specific antibody. Fold induction of phosphorylation was determined by dividing densitometric values of phospho- band to that of total protein bands. Relative abundance of AKT was determined by dividing the densitometric values of AKT to that of beta-actin.

When we looked at the impact of these mutations on insulin-induced activation of AKT, the S668H mutant did not show an effect on the level or phosphorylation of AKT after 5 or 30 min of insulin stimulation, however, the 5 min insulin stimulation lowered the level of AKT in D674H and double mutant expressing cells. Nonetheless, AKT levels recovered after 30 min. insulin stimulation in D674H expressing cells. More importantly, the expression of the double mutant clearly interfered with AKT phosphorylation and reduced the level of phospho AKT below that of wild type IRS1 at both times of insulin stimulation (Figure 2).

**Figure 3:**
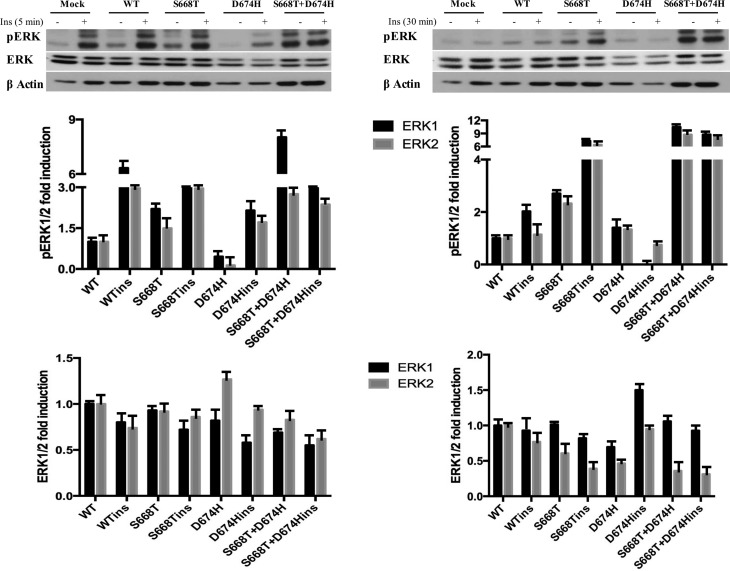
Insulin-induced phosphorylation and expression level of ERK kinase is altered by S668T and D674H mutants in 293T cells. One hundred μg of total protein was fractionated by 10% SDS-PAGE. Blots were labeled with anti-phospho-specific antibodies, stripped and reprobed with non-phospho-specific antibody. Fold induction of phosphorylation was determined by dividing densitometric values of the phospho- band to that of total protein bands. Relative abundance of ERKs was determined by dividing the densitometric values of ERKs to that of beta-actin.

We next looked at the expression and insulin-induced phosphorylation of ERKs. As shown in Figure 3, activation of ERKs by insulin is transient. In cells stimulated with insulin for 5 min, the S668T mutation did not show any effect on background or insulin-induced phosphorylation of ERK1/2, but phosphorylations of ERK1/2 were significantly diminished in cells expressing D674H. However, the phosphorylation of ERK1/2 was prolonged in cells expressing S668H after 30 min of insulin stimulation, and the level of phosphorylation of ERK1/2 dropped below that of control cells in D674H mutant expressing cells. We also observed high level ERK1/2 phosphorylation in cells expressing the double mutant even in the absence of insulin.

**Figure 4:**
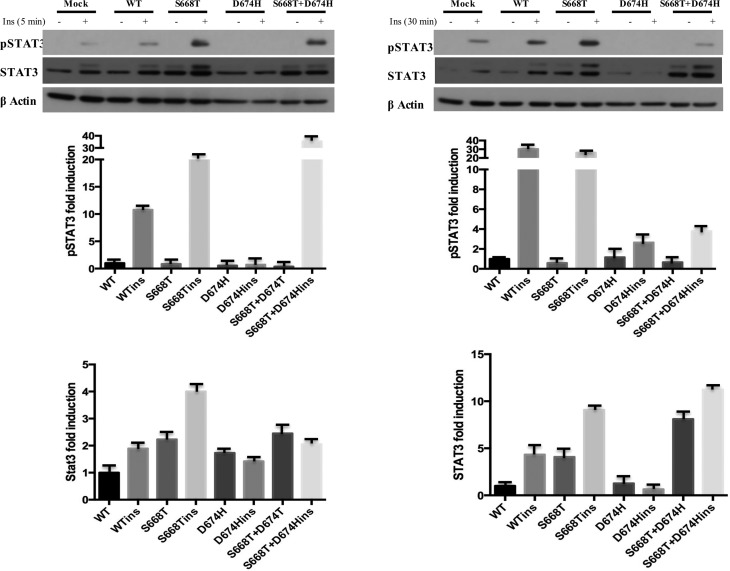
Insulin-induced phosphorylation and expression of STAT3 is altered by S668T and D674H mutants in 293T cells. One hundred micrograms of total protein was fractionated by 10% SDS-PAGE. Blots were probed with anti-phospho-specific antibodies, stripped and reprobed with non-phospho-specific antibody. Fold induction of phosphorylation was determined by dividing densitometric values of phospho- band to that of total protein bands. Relative abundance of STAT3 was determined by dividing the densitometric values of STAT3 to that of beta-actin.

As shown in Figure 4, insulin stimulation for 5 min induced phosphorylation of STAT3 by 10 fold in wild type IRS1 expressing cells, and this was 25 and 35 fold in cells expressing S668T and double mutant, respectively. Although in the absence of insulin we did not see the phospho form of STAT3, shifted STAT3 bands were evident in S668T and double mutant expressing cells. Insulin stimulation for 30 min further increased phosphorylation of STAT3 in wild type and S668T expressing cells, however, the D674H mutation completly inhibited STAT3 phosphorylation by insulin.

### Effects of S668T and D674H on proliferation, migration and glucose uptake

**Figure 5:**
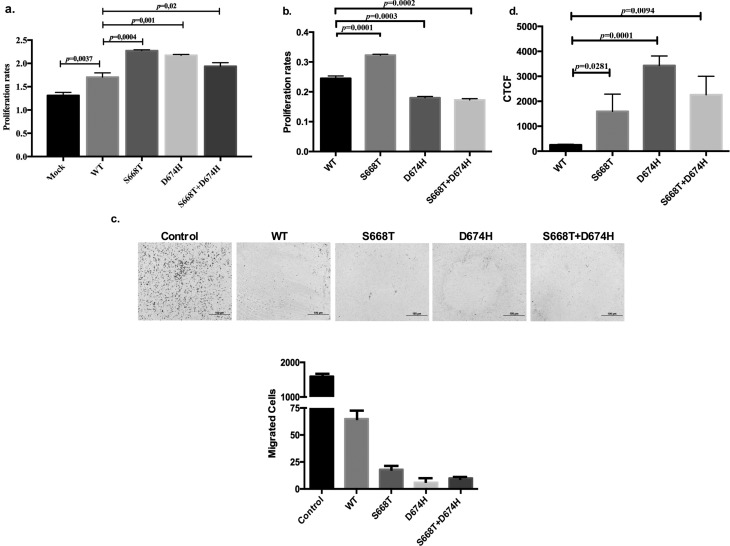
Effects of IRS1, S668T, D674H and double mutants: (a) On proliferation of 293T cells; 293T cells were transiently transfected with expression vectors for 72 h, and cell viability was measured via MTT assay; (b) on proliferation of A549 cells; A459 cells were transiently transfected with expression vectors for 72 h, and cell viability was measured via MTT assay; (c) on migration of 293T cells; 293T cells were transiently transfected with expression vectors for 24 h, trypsinized, counted and 5000 cells were placed in upper chamber BD Biocoat Insert Systems with 8-micron pore size, serum-containing DMEM was added to lower chamber. After overnight incubation, medium in upper chamber was removed, cells were fixed with methanol, and cells in upper chamber were scraped off with cotton swabs. Migrated cells were stained with hematoxylin and eosin. Images of migrated cells were obtained with an Olympus IX51 microscope. (d) Effects of S668T, D674H and double mutants of IRS1 on insulin-induced glucose uptake in A549 cells. A Cayman Glucose Uptake Cell Based Assay Kit was used to determine the level of glucose uptake. (CTCF: Corrected Total Cell Fluorescence)

Since S668T and D674H mutants positively stimulated phosphorylations of IRS1, AKT, ERK1/2, and STAT3 we wanted to determine whether these would affect proliferation of 293T cells. As shown in Figure 5a, transient expression of S668T, D674H and double mutants significantly increased the proliferation of 293T cells compared to wild type IRS1 expressing cells. Since we found conflicting publications about the effect of IRS1 on cancer cell migration (Carvalheira *et al.*, 2003; Li *et al.*, 2013; Porter *et al.*, 2013), we tested our mutants on insulin-induced migration of 293T cells using migration chambers with 8 micron pore size. Mock transfected cells as control group (Figure 5c) showed significant migration in response to serum, and this was inhibited by wild type IRS1. Importantly, our mutants inhibited the migration of 293T cells. Since S668T and D674H mutants differentially affect insulin signaling, we sought to determine whether expression of these mutants would interfere with glucose uptake after insulin stimulation. To address this, A549 cells expressing wild type and mutant of IRS1 were treated with insulin (100 ng/mL) and further incubated for 30 min before glucose uptake was determined. Compared to IRS1 all three mutants significantly increased the uptake of glucose in A549 cells (Figure 5d)**.**

### Effects of S668T and D674H on chemo- and radiosensitivity of cells

**Figure 6:**
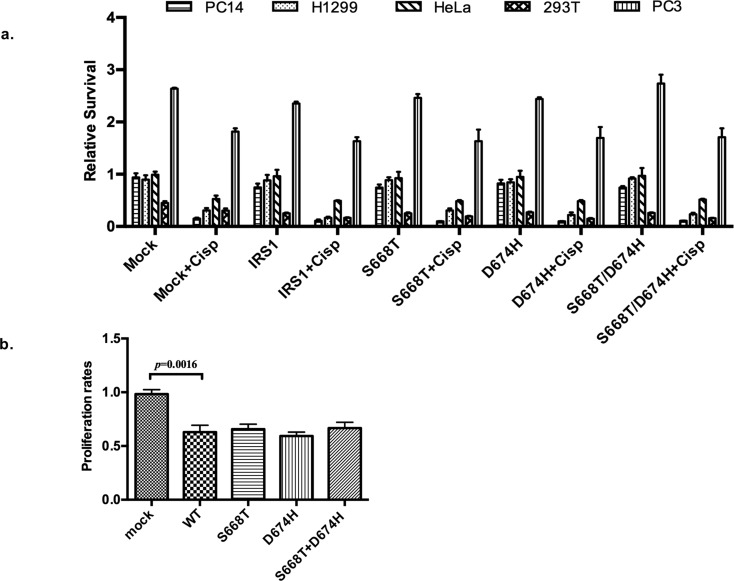
Effects of IRS1, S668T, D674H and double mutants on: (a) Proliferation of cells treated with radiation and cisplatin. A total of 25,000 293T, 10,000 PC14, PC3, HeLa and 8,000 H1299 cells were seeded in 96-well plates, transfected with 300 ng plasmid and 0.4 μL lipofectamine 2000. After 24h, cells were treated with 3 μg/mL cisplatin, and further incubated for 72 hours. For radiation treatment, 293T cells transfected by the same protocol were treated with 5 Gy radiation for 10 min, and further incubated for 48 hours, cell viability was determined with an MTT assay. (Cisp: Cisplatin, PC14: Human lung adenocarcinoma; H1299: Human non small cell lung carcinoma; HeLa: human cervix carcinoma; 293T: human embryonic kidney cells, PC3: human prostate cancer). (b) Effects of novel mutations of IRS1 on radiation-induced death of 293T cells transfected with mutants were measured by MTT analyses.

In recent years, papers have been published about a possible role of IRS1 in chemosensitization (Porter *et al.*, 2012, 2013), however, there is none showing the effect of any mutant of IRS1 in chemo-or radiosensitization. Therefore, we determined whether our mutants would increase or decrease chemosensitization of cancer cell lines, as well as SV40-transformed 293T cells. To test this, 293T, PC14, H1299, PC3, and Hela cells were transiently transfected with wild type and mutants of IRS1. At 48 h after transfection, cells were treated with 3 μg/mL of cisplatin and further incubated for 72 h. As shown in Figure 6a, overexpression of IRS1 sensitized the cells to cisplatin, although the level of sensitivity differed among the cell lines. Compared to wild type IRS1, our mutants did not further sensitize these cells to cisplatin, in fact, S668T mutants reduced the sensitivity of H1299 cells by nearly two fold. After this, we tested the effect of our mutants against radiation-induced growth inhibition of 293T cells. These cells were reverse transfected, as mentioned above, and treated with 5 Gray of radiation for 10 min, and further incubated for 48 h. As shown in Figure 6b, ectopic expression of IRS1 sensitized 293T cells to radiation-induced cell death compared to mock transfected IRS1, our mutants did not increase or decrease radiation sensitivity of these cells compared to wild type IRS1.

## Discussion

Identification of PH, PTB domains, and YXXM motifs in IRS1 made scientists reason that IRS1 could be activated not just by IR but also by other receptors. This questioning produced a significant amount of publications showing activation of IRS1 by GH, VEGF, IGF1, prolactin, and integrins (Vuori and Ruoslathi*,* 1994, Senthil *et al.*, 2002; Gibson *et al.*, 2007; Chan *et al.*, 2008; Porter *et al.*, 2012). Binding of IRS1 to these receptors results in the activation of IRS1. However, with the exception of IGFR1, the above mentioned receptors do not generate signals that control glucose utilization, rather, they induce proliferation and cause cancer (Shaw, 2011). In fact, an association between activation of IRS1 and development of cancer has been established (Vuori and Ruoslathi*.,* 1994; Bergmann *et al.*, 1996; Senthil *et al.*, 2002; Hoang *et al.*, 2004; Koda *et al.*, 2005; Han *et al.*, 2006; Ravikumar *et al.*, 2007), and several pathogenic IRS1 mutations have been identified in lung adenocarcinoma and pancreatic cancer (Carvalheira *et al.*, 2003; Ding *et al.*, 2008; Jiao *et al.*, 2011). However, the functional importance of these mutations has not been elucidated, and for this we studied the functional significance of novel IRS1 mutations identified in NSCLC samples.

Our results indicate that S668T and D674H changes in IRS1 potentiate background and insulin-induced tyrosine phosphorylation of IRS1, although the D674H mutation severely reduced the level of IRS1, and this destabilizing effect was relieved in double mutants. Since IRS1 is degragaded by proteosome-dependent pathway (Zhande *et al.*, 2002), it is highly likely that the D674H mutation may have changed the structure of IRS1 to become a better substrate for proteosome-dependent degredation. Although the D674H mutation increased IRS1 degredation, it induced tyrosine phosphorylation, once again implying a robust structural change in IRS1 protein.

Contrary to what we observed with wild type IRS1, the S668T mutant did not affect phosphorylation of AKT, however total AKT was reduced in D674H expressing cells. Nonetheless, when normalized to total AKT, phospho-AKT appeared to be increased 10 fold. Activation of AKT is induced by double phosphorylation at its activation loop, and phosphorylated AKT is degraded by the proteosome (Adachi *et al.*, 2003). It may be that D674H induced AKT phosphorylation and that this increased its degradation.

When we looked at the effect of our mutants on insulin-induced ERK phosphorylation, the D674H mutant negatively affected both background and insulin-induced ERK phosphorylation, however the S668T mutant showed prolonged activation of ERK. More importanly, the double mutant induced a 12 fold induction of phosphorylation of ERKs even in unstimulated cells. These results clearly indicate that the structural changes in the double mutant of IRS1 significantly potentiated the Grb2-Ras-Erk pathway (Figure 3). In addition to IRS1, AKT and ERK, insulin also induces phosphorylation of the oncogenic transcription factor STAT3 (Carvalheira *et al.*, 2003). When we looked at the effect of our mutants on insulin-induced tyrosine phosphorylation of STAT3, a five minute insulin stimulation induced phosphorylation of STAT3, and the S668T mutant significantly potentiated it, while D674H completely abrogated the insulin-induced phosphorylation of STAT3. The negative effect of the D674H mutant was dominant over the S668T mutation, because in cells expressing double mutant pSTAT3 it almost disappeared at 30 min post insulin stimulation (Figure 4). According to these findings, it is likely that the S668T mutation could inhibit, but the D674H mutation may potentiate binding of Shp2 and regulate the tyrosine phosphorylation level of STAT3.

Since wild type IRS1 and our mutants differentially affect downstream elements of the insulin signaling we wanted to see whether the effects would impact on cell proliferation, migration, glucose uptake, and response to radiation and cisplatin-induced cell death. As shown in Figure 5a, our mutants potently induced proliferation in 293T cells, while only S668T stimulated insulin induced proliferation of A549 cells (Figure 5b). To better understand the molecular mechanism behind the proliferation of A549 cells, we determined the effect of our mutants on insulin-induced glucose uptake and found that all mutants significantly induced glucose uptake in A549 cells (Figure 5d). According to these results, elevated levels of activation of pAKT, pERK, and pSTAT3 in mutant IRS1-expressing cells seem to positively affect cell physiology. In general, IRS1 suppresses, while IRS2 induces the migration of cancer cells (Gorgisen *et al.*, 2017). When we tested wild type IRS1 and our mutants on insulin-induced migration of 293T cells we observed that all forms of IRS1 proteins significantly inhibited migration, although there were some differences in potency (Figure 5c). These results indicate that the mutants still retain characteristic properties of IRS1.

Recently, two papers have been published on the role of IGF1, insulin, and IRSs on chemo-and radiosensitivity (Porter *et al.*, 2012, 2013). Therefore, we tested our mutants on cisplatin and radiation-induced cell death. As shown in Figure 6a, overexpression of IRS1 sensitized H1299, PC14, PC3, HeLa, and 293T cells to cisplatin-induced cell death, and our mutants did not significantly affect this. Moreover, ectopic expression of wild type IRS1 and our mutants sensitized 293T cells to radiation-induced death to the same extent (Figure 6b).

Collectively, we have shown the biological impact of newly identified mutations within the IRS1 gene and suggest that these mutations may be diagnostic markers in lung cancers.

## Supplementary material

The following online material is available for this article:

Figure S1 Representative results for mutational analysis of: (a) IRS1 detected in tumor samples compared to matched controls
