# Supplementary material for: Identification of novel mutations of Insulin Receptor Substrate 1 (IRS1) in tumor samples of non-small cell lung cancer (NSCLC): Implications for aberrant insulin signaling in development of cancer
Source: Genet Mol Biol. 2019 Feb 25;42(1):15–25. doi: 10.1590/1678-4685-GMB-2017-0307 (PMC6428125; doi:10.1590/1678-4685-GMB-2017-0307)
Supplement: Supplementary file 1 [file 1415-4757-GMB-1678-4685-GMB-2017-0307-20190124-suppl.pdf]

Supplementary Material to "Identification of novel mutations of Insulin Receptor Substrate 1 (IRS1) in tumor samples of non-small cell lung cancer (NSCLC): Implications for aberrant insulin signaling in development of cancer"

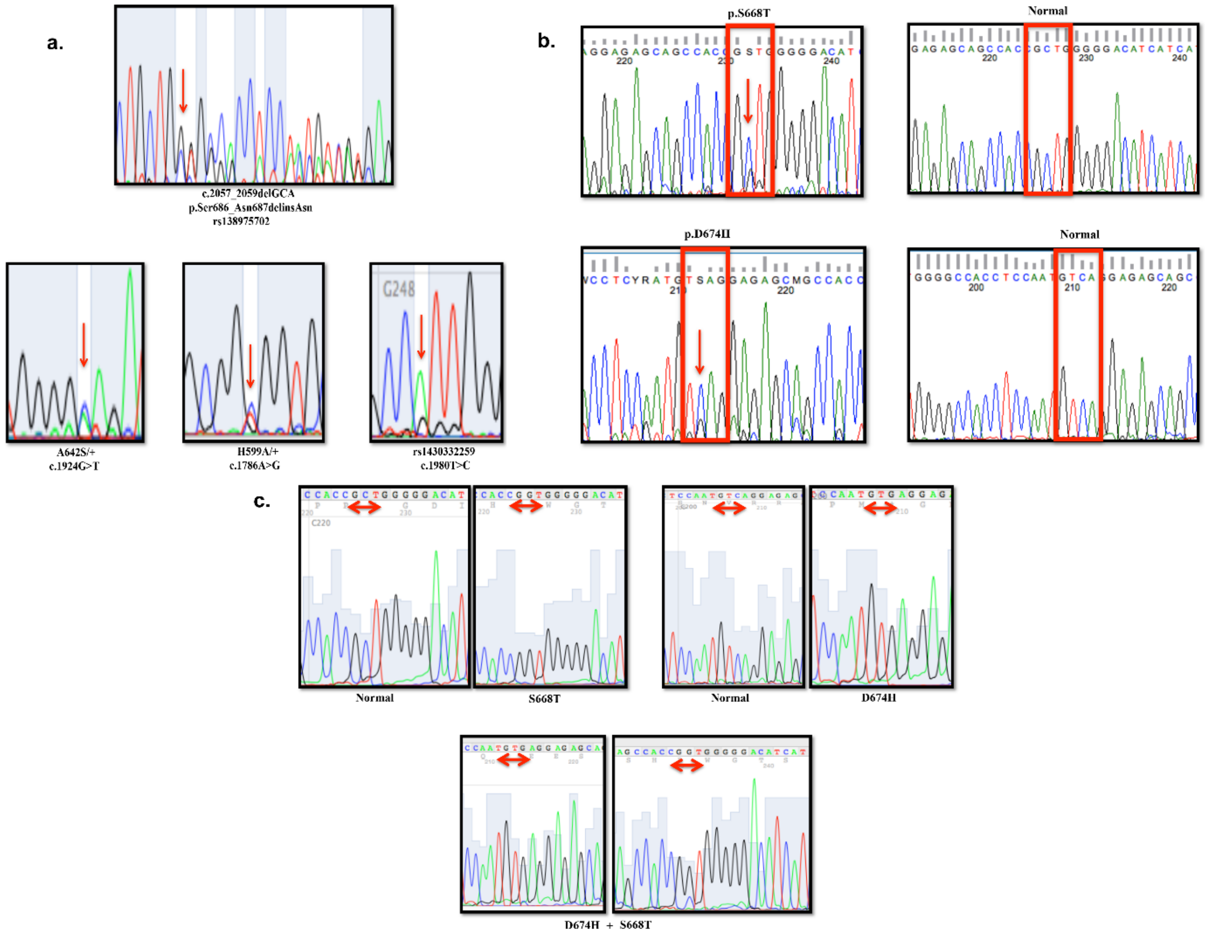

Figure S1 - Representative results for mutational analysis of: (a) IRS1 detected in tumor samples compared to matched controls. These genomic changes were identified in the literature; (b) novel IRS1 p.S668T and p.D674H heterozygote mutations compared to matched controls; (c) generation of novel mutations in pcDNA3 flag-tagged human IRS1 gene using Site Directed Mutagenesis. Mutations were confirmed by Sanger sequencing. Changes in aminoacid codons are indicated by arrows.
